# Ballasting by cryogenic gypsum enhances carbon export in a *Phaeocystis* under-ice bloom

**DOI:** 10.1038/s41598-018-26016-0

**Published:** 2018-05-16

**Authors:** J. E. Wollenburg, C. Katlein, G. Nehrke, E.-M. Nöthig, J. Matthiessen, D. A. Wolf- Gladrow, A. Nikolopoulos, F. Gázquez-Sanchez, L. Rossmann, P. Assmy, M. Babin, F. Bruyant, M. Beaulieu, C. Dybwad, I. Peeken

**Affiliations:** 1 Alfred-Wegener-Institut Helmholtz-Zentrum für Polar- und Meeresforschung, D-27570 Bremerhaven, Germany; 2 AquaBiota Water Research, SE-11550 Stockholm, Sweden; 3 Godwin Laboratory for Palaeoclimate Research, Department of Earth Sciences, University of Cambridge, Cambridge, CB2 3EQ United Kingdom; 4 Norwegian Polar Institute, Fram Centre, 9296 Tromsø, Norway; 5 Takuvik Joint International Laboratory, Université Laval and CNRS, G1V 0A6 Québec, Canada; 6 Université de Sherbrooke, Department of Civil Engineering, QC J1K 2R1 Sherbrooke, Canada; 7 Department of Arctic and Marine Biology, Faculty of Biosciences, Fisheries and Economics, Universitetet i Tromsø - The Arctic University of Norway, N-9037 Tromsø, Norway

## Abstract

Mineral ballasting enhances carbon export from the surface to the deep ocean; however, little is known about the role of this process in the ice-covered Arctic Ocean. Here, we propose gypsum ballasting as a new mechanism that likely facilitated enhanced vertical carbon export from an under-ice phytoplankton bloom dominated by the haptophyte *Phaeocystis*. In the spring 2015 abundant gypsum crystals embedded in *Phaeocystis* aggregates were collected throughout the water column and on the sea floor at a depth below 2 km. Model predictions supported by isotopic signatures indicate that 2.7 g m^−2^ gypsum crystals were formed in sea ice at temperatures below −6.5 °C and released into the water column during sea ice melting. Our finding indicates that sea ice derived (cryogenic) gypsum is stable enough to survive export to the deep ocean and serves as an effective ballast mineral. Our findings also suggest a potentially important and previously unknown role of *Phaeocystis* in deep carbon export due to cryogenic gypsum ballasting. The rapidly changing Arctic sea ice regime might favour this gypsum gravity chute with potential consequences for carbon export and food partitioning between pelagic and benthic ecosystems.

## Introduction

The particulate organic carbon flux from the photic zone provides the major food supply to the seafloor community^1^ and is an important mechanism of atmospheric CO_2_ drawdown but usually less than 1% of primary produced organic carbon reaches abyssal depths^2^. However, excess density by incorporation of ballast minerals (biominerals and lithogenic material) can significantly increase the sinking speed of fresh organic matter and magnitude of carbon export^2–5^. Thus, mineral ballasting plays an important role in strengthening the biological carbon pump that transfers particulate organic carbon (POC) from the surface to the deep ocean^6^. Ballasting by lithogenic material plays a minor role in the deep Central Arctic Ocean^6–8^ and the scarce sediment-trap data available indicate that mineral ballasting of organic carbon in the ice-covered Arctic Ocean is three orders of magnitude lower than the global average^6^. The resulting POC flux to depths >1000 m in the ice-covered Arctic Ocean is usually 0.17–1 gC m^−2^yr^−1^ (refs^9,10^) (Supplementary Table S1), and is considered to be amongst the lowest in the global ocean^6^. Even so, infrequently efficient carbon export events have been observed that were associated with the release of ice algal aggregates^10,11^. These notable exceptions highlight the importance of fast-sinking particles for POC export^12^ in the Central Arctic.

Concurrent with the transformation of the Arctic sea ice from a thick, multi-year to a thinner, first-year ice cover, recent observations have detected phytoplankton blooms beneath snow-covered sea ice early in the season^13^ and below ponded ice during the melt period^14^. The fate of these under-ice blooms is unknown but their occurrence suggests that there is more organic material available for ice-associated mineral ballasting under the new sea ice regime. The recent increase in *Phaeocystis* under-ice^13^ and marginal ice zone^15–17^ blooms in the European Arctic could have a negative impact on the strength of the biological carbon pump since *Phaeocystis* is thought to contribute less to deep carbon export than diatoms^18^. Whether further seasonal and spatial shifts in primary production and alterations in the phytoplankton community composition will influence POC export in the ice-covered Arctic Ocean is still an open question^19–21^.

The precipitation of gypsum (CaSO_4_ · H_2_O) has recently been reported in Arctic sea ice^22^, but this mineral has never been implicated as a potential ballasting material for POC. Here, we report cryogenic gypsum ballasting of an under-ice phytoplankton bloom dominated by the haptophyte *Phaeocystis* north of Svalbard: this is the first report of cryogenic gypsum ballasting.

## Results

**Figure 1:**
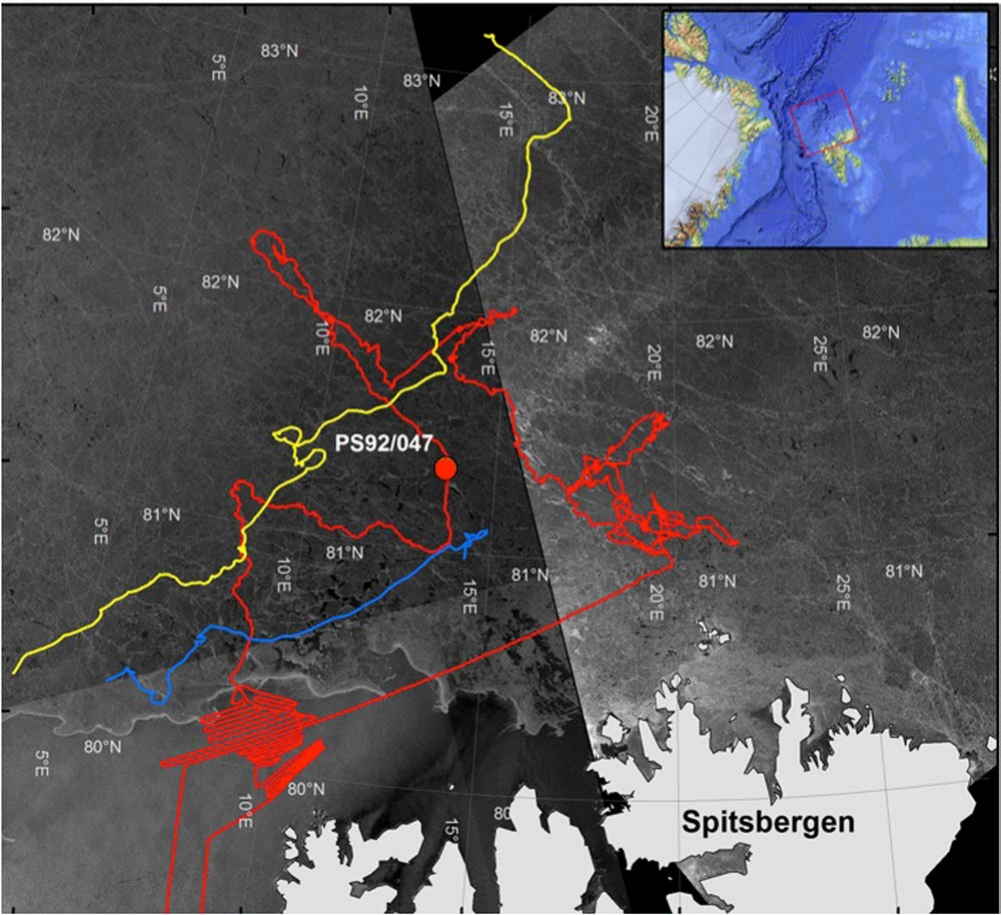
Map of the TRANSSIZ cruise track (red line) and drift trajectories of floes 3 (yellow) and 4 (blue) north of Spitsbergen from the N-ICE2015 campain. The red dot indicates the position of ice station PS92/47 in the Sophia Deep. The background image is a mosaic of radar images from 8 June 2015 (Sentinel-1 Radar Backscatter © ESA; Data Provider: Drift & Noise Polar Services). The map of the study area was created using ArcMap 10.4.1 (Esri) with the standard coastline dataset and bathymetry data from the General Bathymetric Chart of the Oceans (GEBCO)-08 grid, version 20100927, http://www.gebco.net, with permission from the British Oceanographic Data Centre (BODC).

**Figure 2:**
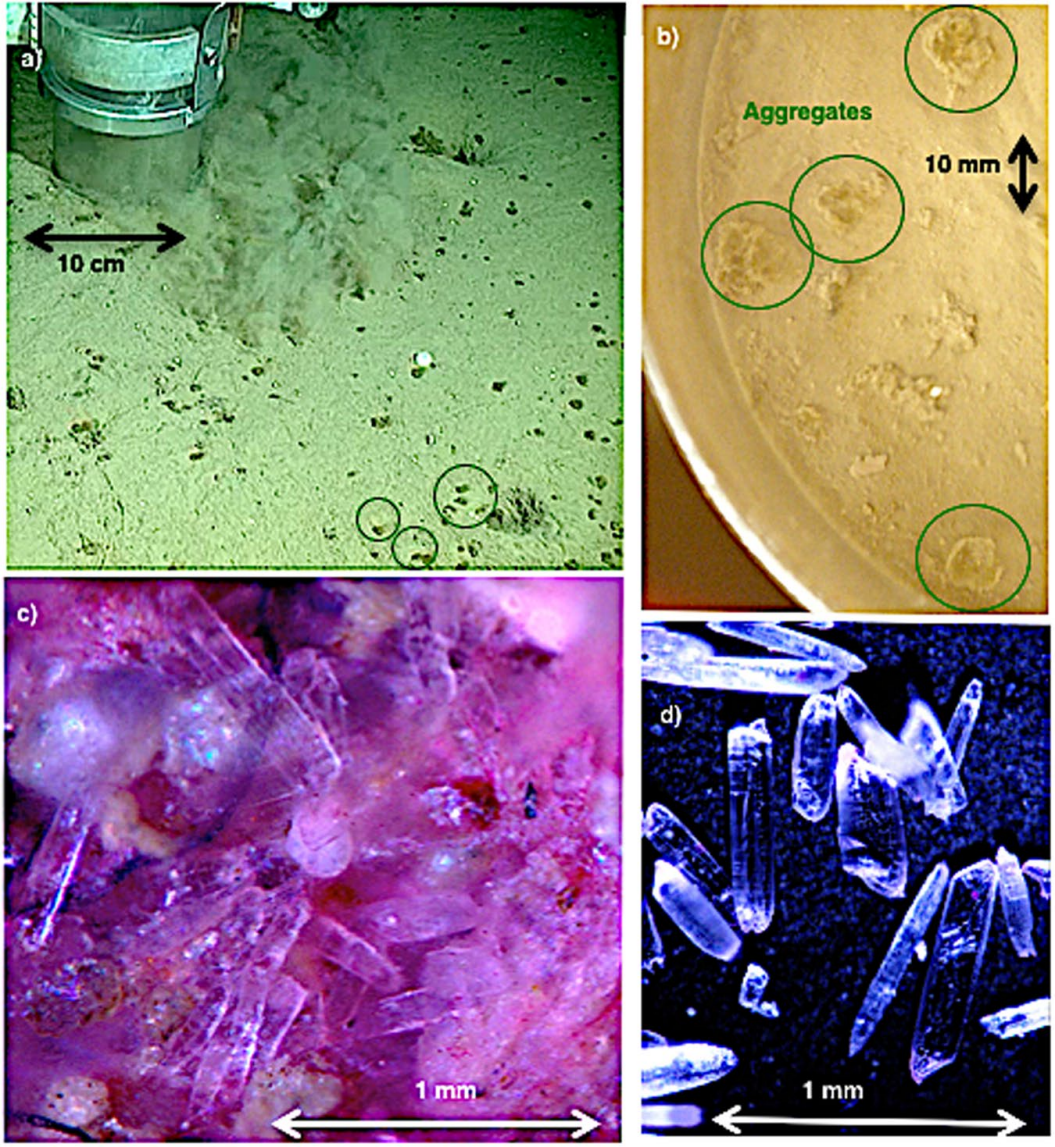
Images of *Phaeocystis* aggregates and associated gypsum crystals. (**a**) *Phaeocystis* aggregates (examples indicated by green circles) observed on the sea floor at 2146 m depth. (**b**) *Phaeocystis* aggregates (green circles) from the top of a multicorer tube surface. (**c**) Gypsum crystals entangled in *Phaeocystis* colonial strands. Remains of *Phaeocystis* aggregates are stained pinkish by the protein stain Rose Bengal^18^. (**d**) Isolated gypsum crystals.

In the spring of 2015, the international “Transitions in the Arctic Seasonal Sea Ice Zone” (TRANSSIZ) expedition of RV Polarstern (PS92, ARK-XXIX/1, 19 May–26 June) systematically investigated the sea-ice ecosystem north of Spitsbergen (Fig. 1)^23^. At Ice Station PS92/47 (81°22′N, 13°36′E, 2146 m; Fig. 1), which was occupied on June 19 and 20, a multicorer (MUC) with eight tubes was used to retrieve sea-floor surface sediment samples. Simultaneously, a video camera mounted on the MUC frame recorded marine snow aggregates throughout the two hours for both the down- and upcast and, while on the sea floor, those that had already settled (Fig. 2a,b, Supplementary Video S1 and Supplementary Fig. S1).

To understand the origin and composition of the marine snow, aggregates from the sediment surface and, subsequently, from the water column at site PS92/47 were collected and investigated. Larger marine snow aggregates were pipetted from the sediment surface of the MUC tubes (Fig. 2a–b) and like sediments of the topmost surface centimetre stored in a Rose Bengal–ethanol solution^24^. In the home laboratory, all samples were washed with tap water over a 63-µm mesh sieve, dried, and examined under a stereo microscope.

The >63-µm residue of the Rose Bengal–ethanol-treated algal aggregates (see methods for details) showed remains of *Phaeocystis* colonies and abundant needle-like crystals: the crystals amounted to half of the aggregate volume (Fig. 2c–d). No biominerals (e.g., planktonic foraminifera, diatom frustules), faecal pellets or terrigenous material were observed in the >63-µm residue (Fig. 2c). However, due to the mesh size that was adapted to micropaleontological work, smaller particles may have been missed during microscopic observations. The needle-like crystals, which had a maximum length of 1 mm and a diameter of 30–300 µm, were only observed in the algal aggregate samples treated with Rose Bengal–ethanol, with none found in the surrounding sediments. No crystals were detected in unpreserved aggregates (stored only in seawater). Thus, for future studies, it is important to note that gypsum crystals were preserved only in ethanol-fixed samples.

To confirm that the *Phaeocystis* aggregates on the sediment surface had settled through the water column, plankton (>55-µm) and aggregates from the water column (in the upper 2000 m) were collected during two Multi Plankton Sampler (MultiNet) casts (see method). Microscopic analyses of the Rose Bengal–ethanol treated, washed, and dried residues confirmed that remains of *Phaeocystis* aggregates with embedded crystals occurred in samples from all water depths. However, quantification of the relative aggregate/crystal number and volume was hampered by the sampling technique itself. The use of MultiNets could have allowed re-aggregation of gypsum crystals and aggregates, as well as loss of an unknown quantity of thin needle-like crystals through the nets. Nevertheless, the finding of needle-like crystals entangled in algal aggregates caught with the MultiNets supports the observation of an export event occurring during the video recording and very recent sedimentation of the algal aggregates retrieved from the seafloor.

The crystals retrieved from the aggregates were identified as gypsum by means of confocal Raman microscopy (see Supplementary Fig. S2). The isotopic signature of the gypsum crystals (δ^34^S = +21.3‰) was consistent with the enrichment expected for abiotic gypsum precipitation from a marine sulphate source (typically δ^34^S = +20.3‰ ± 0.8‰)^25^. This observation indicates that the gypsum crystals were precipitated from marine sulphate within sea ice rather than from oxidation of reduced sulphur in biogenic pyrite or organic matter within the algal aggregates, which would have yielded lower δ^34^S values (−50‰ to +15‰)^26–29^.

**Figure 3:**
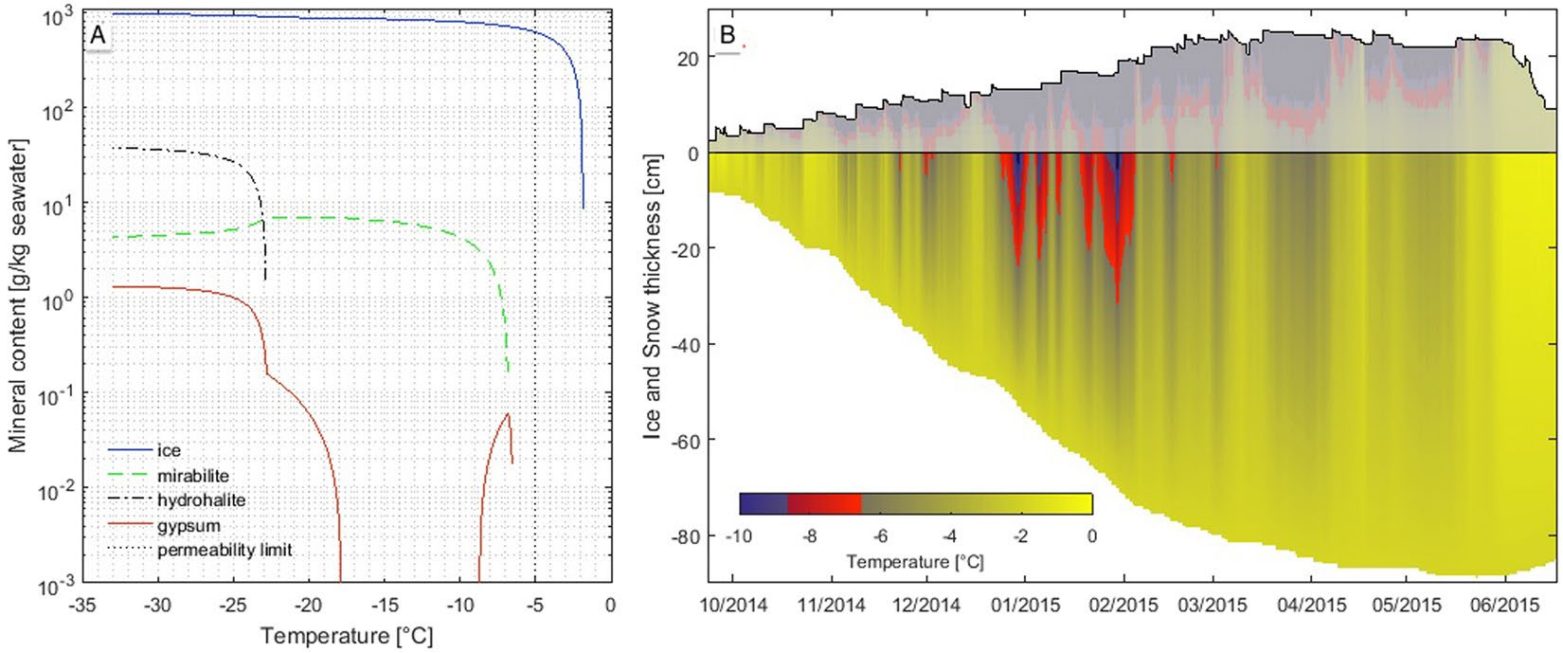
Gypsum formation in sea ice: (**A**) Temperature dependence of the precipitation of gypsum and other minerals during the freezing of standard seawater, as calculated by FREZCHEM^30^. (**B**) Evolution of sea-ice temperature, as modelled by SNOWPACK^58^. The possible window of gypsum precipitation is highlighted by the red part of the colour bar. Snow temperatures are shaded in white to illustrate that no gypsum precipitated from snow.

To estimate the amount of gypsum in the local sea ice, we reconstructed its thermal history and applied FREZCHEM, a chemical–thermodynamic model that was explicitly developed to quantify aqueous electrolyte properties at sub-zero temperatures; it calculates the precipitation of solids by solving the equations of thermodynamic equilibrium using the Pitzer approach^30,31^. We used FREZCHEM to calculate mineral precipitation in high-salinity brines, and found that gypsum can be precipitated between −6.5° and −8.5 °C and below −18 °C (ref.^30^) (Fig. 3). Temperatures in first-year Arctic sea ice never reach as low as −18 °C; thus, only the narrow ‘warmer’ precipitation range is of interest in this study. Our model calculations indicate that up to 2.7 g gypsum m^−2^ could be precipitated. This value is comparable to the observed *Phaeocystis* (1.6 g C m^−2^) and POC (9.4 g m^−2^) standing stocks at Station PS92/47 (Supplementary Table S2) and to standing stocks found during the contemporaneous Norwegian young sea ICE (N-ICE2015) expedition that operated in the vicinity of the current study (average 1.3 g *Phaeocystis* C m^−2^, average 11.1 g POC m^−2^; Supplementary Table S2). The ballasting of POC (here *Phaeocystis* carbon) with 50% gypsum would increase the density from ~1 to 1.65 g cm^−3^ with gypsum ballasting. This density increase could enhance the amount and speed of surface-derived organic carbon export to the abyssal ocean^32^.

The thermodynamic FREZCHEM^30^ model does not account for the time scales of gypsum dissolution outside the stability range. The direct observation of gypsum crystals at a depth of more than 2 km presented here indicates that the relatively large gypsum crystals observed were stable enough to survive the descent through the water column. Even so, our current knowledge of the kinetics of gypsum dissolution under the conditions at the sampling site is insufficient to exclude the possibility that some smaller crystals dissolved during the descent. Gypsum crystals might have been expelled from the sea ice during warming, even before melting, when brine pockets interconnect at T > −5 °C (ref.^33^), which is near the warm gypsum precipitation window. The release of crystals to the water column coincided with a large *Phaeocystis* bloom (Supplementary Fig. S3) that developed beneath the dense pack ice (1.4 m modal sea ice thickness), despite the heavy snow cover (0.4 m modal snow thickness), 80 km north of the marginal ice zone (Fig. 1). The under-ice bloom was facilitated by light transmission through leads in the ice pack and could be observed for one month, from 25 May until 22 June^13^.

## Discussion

We report here for the first time on the ballasting of a *Phaeocystis* bloom by cryogenic gypsum released from melting sea ice and thus representing a previously unknown role of this mineral in enhancing deep carbon export in ice covered areas of the world ocean.

The genus *Phaeocystis* is a common member of the phytoplankton community in the marginal sea-ice zone^15,34^. *Phaeocystis* is associated with the inflow of Atlantic water into the Arctic Ocean^16,34^. It is assumed that the higher temperature of the Atlantic water promotes blooms dominated by *Phaeocystis* in the eastern Fram Strait^34,35^. The ability of *Phaeocystis* to bloom under low and changing light conditions^36^ enables this organism to take advantage of the occurrence of leads opening up between ice floes^13^. The combination of a relatively shallow mixed layer (Supplementary Fig. S3) and an occasional light supply through the leads allows *Phaeocystis* to bloom relatively early and far north (north of 81°N)^13^.

Although *Phaeocystis* has been found in surface sediments in the Southern Ocean^37^, it has been proposed that this alga is remineralized within the upper 50–100 m (refs^38,39^). However *Phaeocystis* export events have recently been observed below 300 m at the ice edge in the Arctic Ocean^17,40^, but they were not related to a release of gypsum crystals and thus did not demonstrate the ballasting export mechanism presented here. *Phaeocystis* can form large blooms in its colonial stage^36^. In *Phaeocystis* colonies (which reach sizes of up to 2 mm), the cells are embedded in a matrix of polysaccharides – they are known to excrete large amounts of mucopolysaccharides and heteropolysaccharides^36^. This combination of large size and the sticky polysaccharide gel matrix^38,39,41^ make *Phaeocystis* colonies in the water column an ideal trap for gypsum crystals released from sea ice. Our video recording of the MUC deployment and aggregate accumulation on the sea floor clearly indicates downward flux of the gypsum-ballasted *Phaeocystis* (Fig. 2 and Supplementary Data Video S1). Given the timing of the bloom in the study area^13^, we infer that this process occurred within a relatively short time.

Backtracking the ice floe^42^ beneath which we observed the *Phaeocystis* export event, we calculate that gypsum crystals must have formed between December 2014 and February 2015 at temperatures below −6.5 °C. The gypsum crystals would have been released shortly before our sampling operations. This scenario is supported by the simultaneous occurrence of sea-ice temperatures above −5 °C (ref.^23^, fig. 7.2.2), abundant particles in the water column, and gypsum-ballasted algal aggregates at the sediment surface at a depth of 2146 m, as observed at station PS92/47 (Fig. 2, Supplementary Video S1 and Supplementary Fig. S1). Temperature-induced increase in the brine-channel size and connectivity potentially facilitated the release of gypsum crystals into the water column, coincident with the presence of a similar mass per area of *Phaeocystis*.

To illustrate the effect of this potential ballasting on carbon drawdown from the surface, we estimated the potential maximum carbon drawdown by *Phaeocystis* using the integrated net primary production (NPP) for the upper 50 m at station PS92/47. The total NPP was 0.24 ± 0.02 g C m^−2^ d^−1^, with a *Phaeocystis* contribution of 56% (estimated via the share of total phytoplankton carbon standing stock) corresponding to a daily production share of 0.13 ± 0.02 g C m^−2^ d^−1^ (Supplementary Table S2). Based on a bloom duration of at least 28 days^13^, the estimated *Phaeocystis* NPP amounted to a total of 3.8 ± 0.2 g C m^−2^ (Supplementary Table 2). Although no shallow carbon export was measured in this study, a purely speculative carbon export of only a quarter of the above-estimated *Phaeocystis* NPP (approximately 1 g C m^−2^) to the seafloor during the short-term gypsum ballasting export event would rival the annual carbon export in various deep-sea regions of the Arctic Ocean (Supplementary Table S1). This is in accordance with previous observations that rapid ballasted-particle export can be extremely efficient over very short time scales^12^.

Given the ongoing changes in the prevailing ice type (thin, first-year sea ice replacing thicker multi-year ice)^19^, the annual release of cryogenic gypsum may increase in the future. The apparent increase of *Phaeocystis* blooms in the European Arctic^16^, the prevalence of under-ice phytoplankton blooms^13,14^, and the rapid melting of gypsum-containing sea ice could strengthen the export of phytoplankton, particularly *Phaeocystis*. With the anticipated increase in Arctic primary production^43^, this ballasting process could lead to an increase in the food supply for abyssal organisms. This change in carbon export efficiency from the surface ocean could impact the structure and functioning of benthic ecosystems, and subsequently alter the cycling of carbon and other biogenic elements, especially in the ice-covered Arctic Ocean. However, this hypothesis still has to be tested in future studies and will require a dedicated observational programme that includes targeted sampling of sea ice and the surface mixed layer, as well as deployments of free-floating sediment traps at various depths for extended time periods.

## Methods

Unless stated otherwise, all treatments, measurements and analyses were carried out at the Alfred-Wegener-Institut Helmholtz-Zentrum für Polar- und Meeresforschung in Bremerhaven, Germany.

### Video-equipped multicorer

A multicorer (MUC) with eight tubes of 10-cm inner diameter was used for retrieving undisturbed sea-floor surface sediment samples. Simultaneously, a Sanyo HD400P video camera (10× optical zoom, autofocus, 330 kbit s^−1^) mounted to the MUC frame routinely recorded the descent and ascent of the MUC (Supplementary Video S1 and Supplementary Fig. 1).

As we had no idea that we would encounter cryogenic gypsum our sampling strategy and applied methods followed the micropaleontological protocol for benthic foraminifera investigations.

Larger marine snow aggregates approximately 1 cm in diameter were pipetted from the sediment surface of three of the MUC tubes selected at random (Fig. 2a,b). The aggregates were transferred into a container with a sample-equivalent volume of Rose Bengal–ethanol solution^24^. Hereby, ethanol hampers the disintegration of organic matter and gypsum, Rose Bengal stains proteins of early aggregate invaders like foraminifers. In addition, the topmost surface centimetre of each of the three selected MUC tubes was similarly preserved.

### Multi plankton sampler

A Midi Hydro-Bios MultiNet, Kiel, Germany (MultiNet), with five nets, each with an opening of 50 × 50 cm, a length of 250 cm and a 55-µm mesh was used for plankton sampling. At station PS92/47 the water column was sampled above 100, 200, 600, 1000 and 2000 m water depths at a speed of 1 m s^−1^.

### Treatment of aggregate and MultiNet samples

In the home laboratory, all samples were washed with tap water over a 63-µm mesh sieve, dried at room temperature (MUC samples) or 50 °C (MultiNet samples), and examined under a stereo microscope (100–160× optical magnification).

### Pigment analyses

For pigment analysis with high pressure liquid chromatography (HPLC), seawater samples (1–2 L) were taken with Niskin bottles from seven depths in the upper 100 m attached to a rosette water sampler with a package of electronic instruments (SBE911plus) that continuously measures conductivity, temperature, and depth (CTD). The sample handling and pigment measurement processing were carried out as described in Kilias *et al*.^44^. The taxonomic structure of the phytoplankton groups was calculated from marker pigment ratios using the CHEMTAX program^45^. Pigment ratios were constrained as suggested by Higgins *et al*.^46^ based on microscopic examination of representative samples during the cruise, and the input matrix published by Fragoso *et al*.^47^ was applied. The resulting *Phaeocystis* contribution represents a percentage of the total chlorophyll *a* (Chl a) biomass and is expressed as µg Chl a L^−1^. The standing stocks of *Phaeocystis*, in terms of carbon biomass, were calculated by multiplying the chlorophyll concentrations with a conversion factor of 31.4, which was estimated for this bloom by Assmy *et al*.^13^.

### Microscopic analyses of phytoplankton

To verify the HPLC pigment measurements, the phytoplankton taxonomic composition was analysed by light microscopy in samples from the chlorophyll maximum (shown for station PS92/47 in Supplementary Fig. 3d). Seawater samples were preserved in hexamethylenetetramine-buffered formalin (final concentration 0.5%) and stored in amber glass bottles. For the microscopic analyses, 50-mL aliquots were transferred to settling chambers where the phytoplankton cells were allowed to settle for 48 h. At least 500 cells of the dominant phytoplankton species or groups were counted with an inverted microscope^48^ using phase contrast and at three different magnifications. Phytoplankton cells were identified to the genus level, and the phytoplankton carbon content was obtained by multiplying the counts by the carbon values for individual cells. The phytoplankton carbon content was calculated as described in Edler^49^.

### Primary production

To calculate photosynthetic parameters, seawater samples from the Niskin bottles were incubated at different light levels in the presence of ^14^C-labelled sodium bicarbonate using the method after Lewis and Smith^50^. To calculate the integrated primary production, the photosynthetically available radiation (PAR) just below the sea ice was derived from the incoming global radiation measured every 10 minutes over a period of 24 hours by the Polarstern weather station. Hourly PAR values were then estimated by integrating the measurements taken during each hour (n = 6). For each hour, the PAR at different depths (2.1, 5.0, 10.0, 15.0, 30.0, and 50.0 m) was estimated using a 4% transmittance through snow and sea ice, as determined from radiometric measurements taken by a remotely operated vehicle (ROV) and the attenuation coefficient (kdPAR) of 0.15 m^−1^, which was estimated from vertical light profiles measured during the time at ice station PS92/47.

The hourly primary production at each depth was then estimated using fitted photosynthetic parameters that were derived from photosynthesis versus irradiance (P/I) curves by fitting the model of Platt *et al*.^51^ in order to account for photoinhibition. The non-linear fitting was done using the Levenberg–Marquardt algorithm from the minpack.lm R package^52^.

At each depth, primary production was estimated using photosynthetic-parameter and PAR estimates. Finally, daily primary production (PP) was calculated by integrating production over time and depth. For error estimation of PP, a Monte Carlo procedure was used to propagate the uncertainty of the fitted photosynthetic parameters on the estimated daily primary production. At each depth, a total of 10,000 simulations (i.e., P/I curves) were performed by randomly sampling parameter values based on a multivariate normal distribution of the fitted parameters. Using the generated curves, the depth and daily integrated primary production rates were calculated as described above. The standard deviation of the 10,000 integrated daily primary production rates was then used as a measure of the uncertainty around the estimated value of primary production.

### POC analysis

Seawater samples for suspended POC were collected from eight discrete depths between 0 and 200 m, with Niskin bottles mounted on a rosette water sampler with a CTD system (SBE911plus). Triplicate samples (300–500 mL depending on concentration) were filtered onto glass microfiber filters (GF/F) filters (Whatman), precombusted at 500 °C, and immediately frozen at −20 °C for laboratory analysis at UiT, the Arctic University of Norway, Tromsø, which occurred within 6 months of sample collection. For analysis, the frozen filters were dried at 60 °C for 24 h and subsequently placed in an acid fume bath (concentrated hydrochloric acid) for 24 h to remove all inorganic carbon. The filters were then placed into a 60 °C desiccator for an additional 24 h and finally put into nickel capsules for analysis. The samples were analysed for POC using an Exeter Analytical CE440 CHN elemental analyser.

### Raman spectroscopy

Single crystals were measured with a confocal Raman microscope (WITec alpha 300 R) using an excitation wavelength of 488 nm (pinhole size: 50 µm) and a 20× (Zeiss EC Epiplan, Numerical Aperture = 0.4) lens. This type of instrument is ideal for unambiguous determination of the mineral phase of small (few µm) crystals^53,54^. As seen from the Raman spectrum (Supplementary Fig. 2), the crystals extracted from the MUC sample yield an exact match to the gypsum in-house standard when measured under identical conditions.

### Sulphur isotopes

The sulphur isotopic composition (δ^43^S) of the gypsum extracted from the MUC samples was measured in the Godwin Laboratory at the University of Cambridge (United Kingdom). Gypsum microcrystals (~300 mg) were handpicked and combusted in a tin capsule in the presence of V_2_O_5_ at 1030 °C by a Flash Elemental Analyser (Flash EA, Thermo Scientific). The sulphur dioxide produced was measured by continuous-flow gas source isotope ratio mass spectrometry (Thermo Scientific, Delta V Plus). The sample run was bracketed by three standards (NBS-127; +20.3‰). A blank (no sample) was analysed before and after the gypsum sample and the block of standards, to avoid a memory effect. No drift was detected during the run. The reproducibility of the method was better than 0.1‰ (1 sd) based on the analysis of the six NBS-127 standards. All δ^43^S values are relative to V-CDT (Vienna-Canyon Diablo Troilite). Consistent with Thode *et al*.^55^ and Pierre^56^, the sulphur isotope value (δ^43^S) of +21.3‰ determined for the gypsum is regarded as indicating a marine source of sulphur (+20.3‰), with an offset of approximately 1‰ due to fractionation during precipitation.

### Weight and volume of gypsum in *Phaeocystis* aggregates

The weight of the gypsum crystals was calculated from a 3-mL volume of algal aggregates pipetted from the MUC sediment surface that was stored in the Rose Bengal–ethanol solution. In the laboratory, the 3-mL sample of algal aggregates was sieved through a 63-µm mesh screen, and the residue was dried at room temperature. All gypsum crystals were picked from this residue, and the weight was determined with a high-precision Sartorius SE2 ultra-microbalance. Using a density value of 2.32 g cm^−3^, gypsum comprised approximately half of the algal aggregate by volume (>600 µg mL^−1^).

The incorporation of gypsum in the *Phaeocystis* aggregates increased the mass density due to ballasting, i.e., 50% (by volume) *Phaeocystis* (density φ ~ 1) plus 50% gypsum (density φ = 2.3) is equivalent to φ~1.65 g cm^−3^.

### Calculation of potential gypsum precipitation

To evaluate the potential for gypsum precipitation in sea ice and quantify the amount of precipitated crystals, we combined ice-floe backtracking, reanalysis forcing data, a thermodynamic sea-ice model and a geochemical freezing chemistry model. Although each of the individual models has shortcomings and uncertainties, we are confident that we achieved the best possible, conservative estimate of precipitated gypsum mass. Solid precipitation in sea ice is mostly driven by temperature evolution. To reconstruct gypsum growth, we reconstructed ice temperatures of the surveyed area over the winter season before sampling. As no ice-mass-balance buoy was within a reasonable distance during period before sampling, ice temperatures were reconstructed using a thermodynamic model forced by reanalysis data. To assess the correct location of the surveyed ice floe throughout the winter, we used a Lagrangian backtracking algorithm employing a variety of sea-ice drift products^42^. This process provided a trajectory of the surveyed ice parcel from its initial formation to the location where it was sampled. This trajectory was used to extract the appropriate temperature and six-hourly meteorological forcing data from the European Centre for Medium-Range Weather Forecasts (ECMWF) ERA-Interim reanalysis product^57^. Together with the assumption of a constant ocean heat flux of 5 W m^−2^, these data were used to force the SNOWPACK thermodynamic model^58–60^ in a newly developed variant SNOWPACK SeaIce Branch model (http://models.slf.ch/p/snowpack/) to derive the ice growth and the internal temperature field. The total ice thickness of 95 cm (sea ice plus superimposed ice/snow ice) calculated by the model is in good agreement with the modal ice thickness of 90–100 cm measured by electromagnetic induction sounding (GEM2, Geophex, Canada) that was provided in the expedition report^23^. The fact that the modelled and observed ice thicknesses correspond well strongly suggests that this approach is suitable for reconstruction of the thermodynamic evolution of the ice parcel, despite all the uncertainties involved in backtracking and reanalysis data^42^.

To calculate the potential for gypsum precipitation, we used the FREZCHEM freezing chemistry model^30^. This model is frequently used for calculations of seawater freezing, and is thus the best tool for an absolute estimate of total precipitated gypsum mass. Seawater freezing was calculated at 0.1 °C intervals for standard seawater, starting at 0 °C. As the precipitation kinetics of gypsum are largely unknown, especially at low temperatures, we had to base our calculations on two assumptions: first, the fact that we found sea-ice-precipitated gypsum in algal aggregates on the deep-sea floor proves that, once they have formed, gypsum crystals remain stable during the melting phase of sea ice. Hence, once gypsum is produced, it will not be dissolved by further thermal changes in the ice cover. Second, we assume that gypsum precipitation occurs immediately when the temperature threshold is reached. As almost nothing is known about the kinetics of gypsum formation, and the FREZCHEM model does not offer a temporal dimension, this is the only suitable assumption. Earlier observations^22^ that gypsum crystals form within a limited time frame support the validity of this assumption.

By adding the maximal gypsum formation potentials of all modelled horizontal layers, we estimated the potential gypsum precipitation on our surveyed ice parcel. To account for brine rejection during sea-ice formation, the final precipitated gypsum masses (of 18.8 g gypsum m^−2^) were divided by the ratio of the seawater salinity of approximately 35 and the observed sea-ice bulk salinity of 5. This results in an estimate of potential gypsum precipitation to 2.7 g gypsum m^−2^.

Overall, our salinity-corrected estimate likely provides an underestimation, as gypsum precipitation was observed by Geilfus *et al*.^22^ within a much wider temperature window, starting at temperatures as high as −3 °C. Higher temperature windows for gypsum precipitation can be easily achieved in FREZCHEM simulations with minor changes to the initial ion composition of the seawater. Additionally, the reanalysis data tend to overestimate snow accumulation on Arctic sea ice, as a significant amount of snow is lost in open leads during snow drift events. Thus, ice temperatures low enough for gypsum precipitation might be present in an even larger portion of the sea-ice column.

### Ocean Data View (ODV)

The data shown in Supplementary Fig. 3 were plotted with the Ocean Data View software^61^. The stations included in the sections are outlined by the red rectangle in the map (c). The plot for *Phaeocystis* (a) is based on the samples from discrete depths in the upper 50 m (see pigment analysis section) and is gridded with the weighted average method in ODV. The temperature (a, b) and salinity (b) contours are based on CTD sensor data^23^ with 1-m vertical resolution and are gridded with the DIVA method in ODV.

## Electronic supplementary material

Supplementary video 1

Dataset 1
