# Supplementary material for: Ballasting by cryogenic gypsum enhances carbon export in a Phaeocystis under-ice bloom
Source: Sci Rep. 2018 May 16;8:7703. doi: 10.1038/s41598-018-26016-0 (PMC5956002; doi:10.1038/s41598-018-26016-0)
Supplement: Supplementary file 2 — Dataset 1 [file 41598_2018_26016_MOESM2_ESM.docx]

**Ballasting by cryogenic gypsum enhances carbon export in a *Phaeocystis* under-ice bloom** Wollenburg, J.E.^1*^; Katlein, C.^1^; Nehrke, G.^1^; Nöthig, E.-M.^1^; Matthiessen, J.^1^; Wolf-Gladrow, D.A.^1^; Nikolopoulos, A. ^2^; Gázquez-Sanchez, F. ^3^; Rossmann, L.^1^; Assmy, P.^4^; Babin, M.^5^; Bruyant F.^5^; Beaulieu, M.^6^; Dybwad, C.^7^; Peeken, I.^1^.

**Supplementary Data**

**Supplementary Video 1:** Example video sequences from the MUC cast at station PS92/47 showing the *Phaeocystis* export event at depths of 50 m, 300 m, 600 m, 1000 m, 1300 m, 1600 m, 1900 m and seafloor. Shackle width is approximately 4 cm diameter.


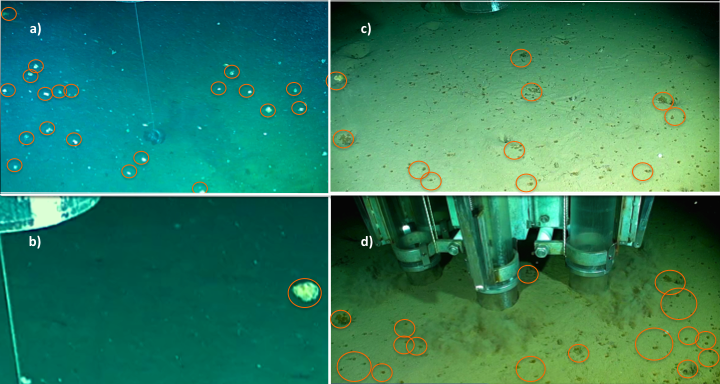


**Supplementary Fig. S1:** Photos from TV-MUC cast 2 m (a & b), and 0.2 m (c) above seafloor, and during coring (d). Large aggregates or accumulations of aggregates are indicated by orange circle. The whitish appearance of aggregates in the water column (a & b) results from the bright searchlights directed bottomwards. A more correct colour reproduction can be achieved close to the seafloor because the light is scattered by the sediments there (Figs. 3c & 3d). Photos are frame captures from the video recording of the TV-MUC system taken by Jutta Wollenburg.


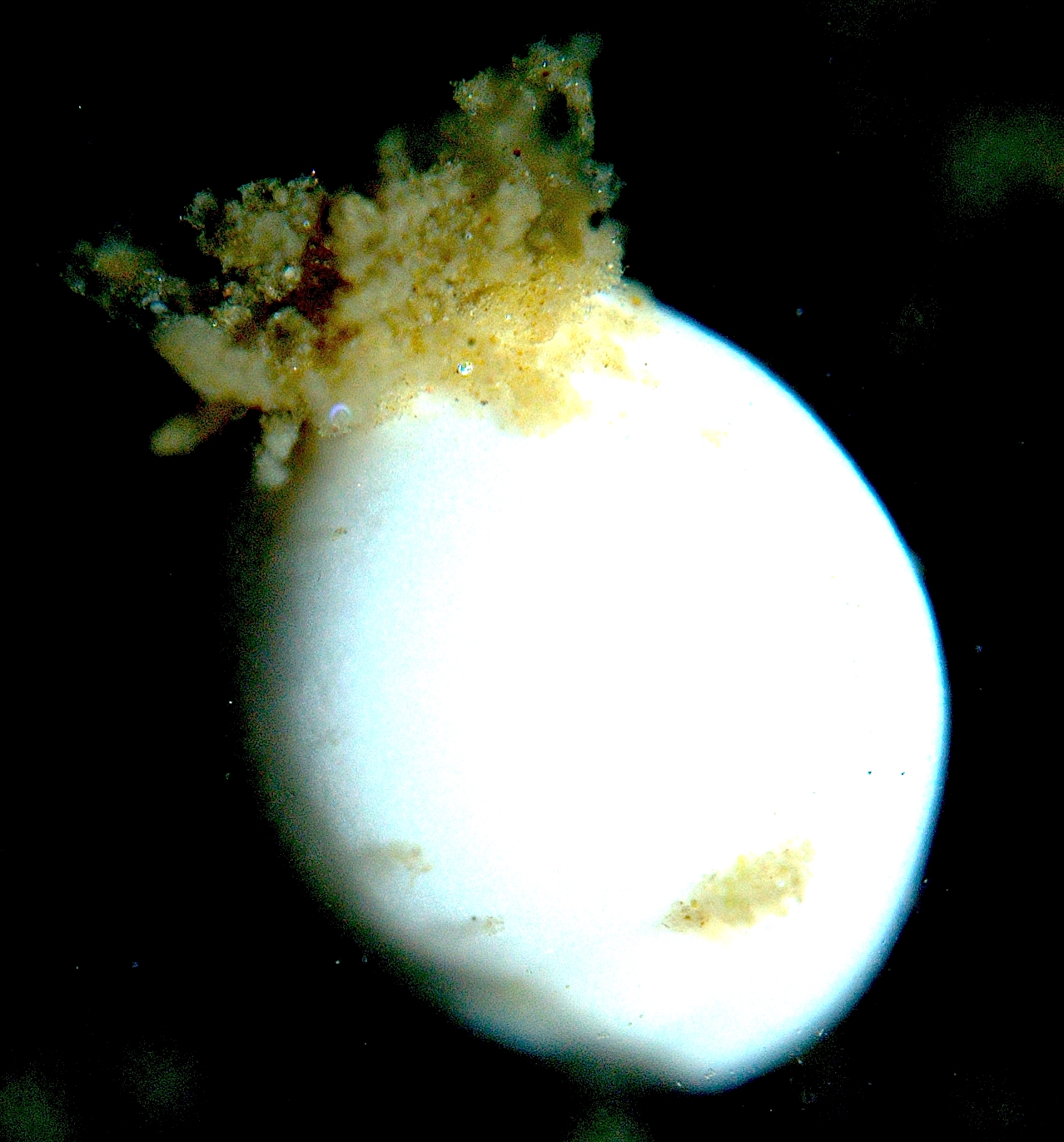


**1 mm**

**Supplementary Fig. S2:** Colonial strands of *Phaeocystis* just being ingested by deep-sea foraminifera *Pyrgo* *rotalaria*.

**
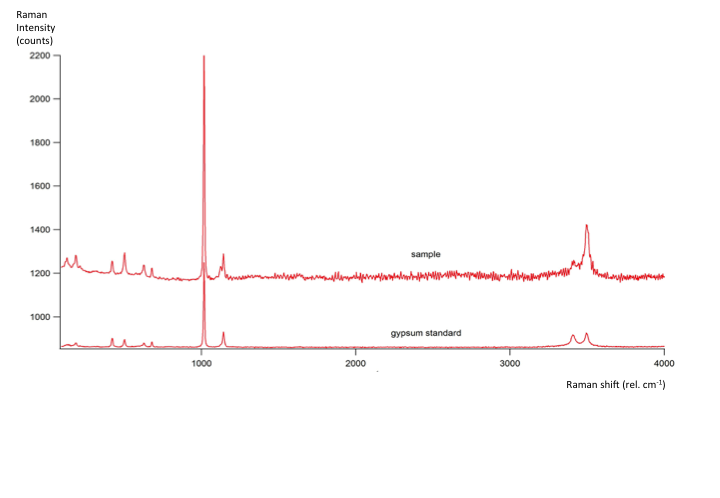
Supplementary Fig. S3:** Raman spectrum of gypsum crystals isolated from *Phaeocystis* aggregates pipetted from the sediment surface at station PS92/47.


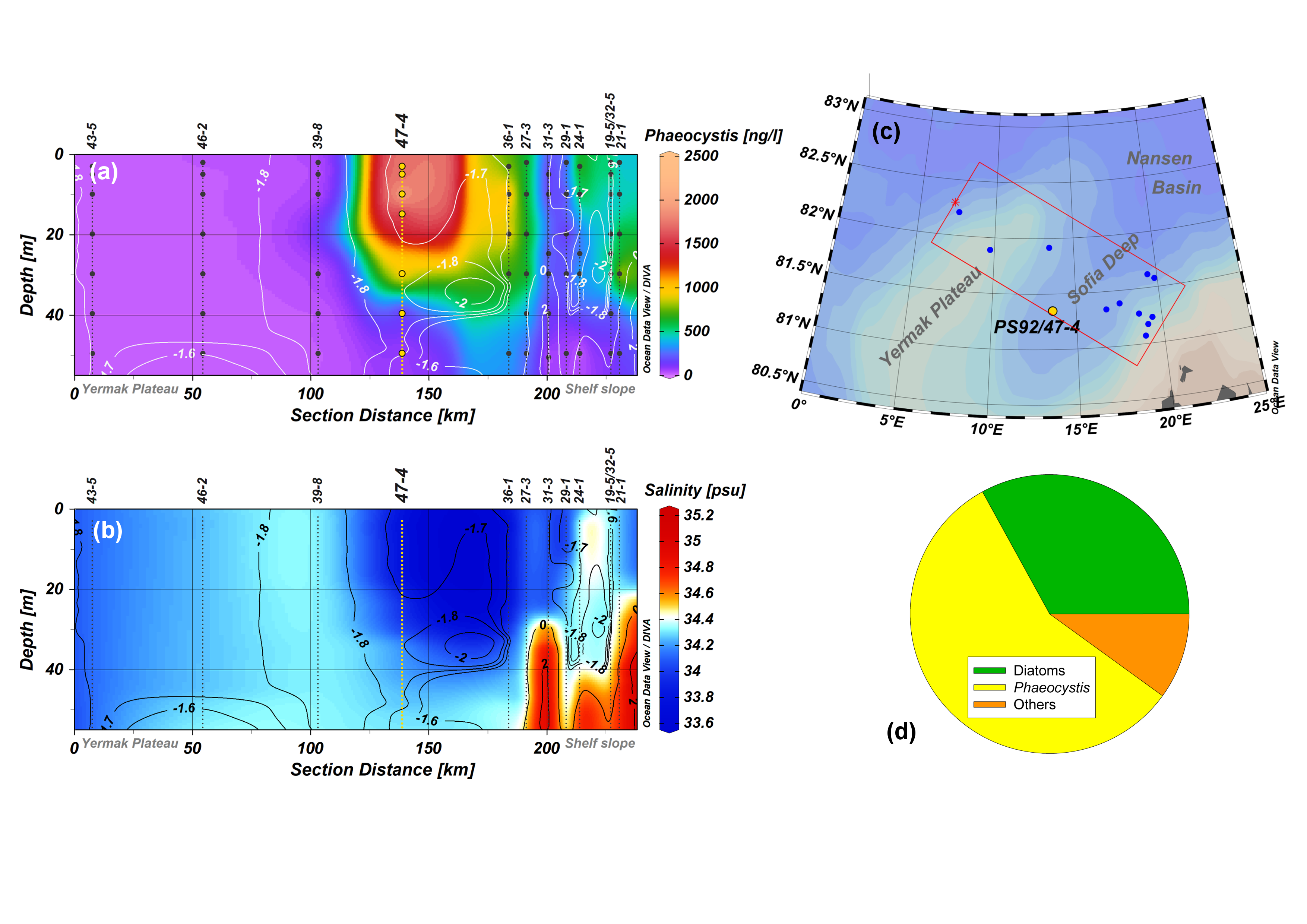
**Supplementary Fig. 3:** Section plots for the upper 50 metres of (a) distribution of *Phaeocystis* Chl-a, and (b) salinity. Both plots are overlaid by contours of potential temperature (in ^°^C; a, solid white; b, black lines). Black dots (yellow for station PS92/47) indicate the sample depths at each station. All section plots comprise data from PS92 stations within the red rectangle shown in the map (c). The section stretches from the Yermak Plateau in the north-west to the Barents Sea continental shelf in the south-east. The yellow dot marks station PS92/47 in the Sophia Deep while the red star indicates the beginning (0 km) of the section over the Yermak Plateau. Note that the displayed order in the section is not always in consecutive order of time. Data are plotted with Ocean Data View software, v4.7.6^61^. (d) Pie chart showing the composition of the autotroph biomass at station PS92/47 (10 m water depth) in carbon per litre based on microscopic counts. Others are mainly consisting of the phototrophic dinoflagellate *Polarella* *glacialis* and the ciliate *Myrionecta rubra*.

**Supplementary Table 1:** Carbon flux in the Arctic Ocean; selection of data obtained by means of sediment traps, ^234^Th-derived, nitrogen budget calculated, and taken from the review of Harada et al.^9^. Values are given as annual flux (g C m^-2^ yr^-1^) and daily flux rates (g C m^-2^ d^-1^). For comparison, the here described carbon export event was estimated to be between 0.81 - 6.18 g C m^-2^ for this single event, depending on the *Phaeocystis* contribution of 12-92%^13^.

| **Method/Region** | **Annual flux** | **Daily flux ranges** | **References** |
| --- | --- | --- | --- |
|  | **g C m^-2^ yr^-1^** | **g C m^-2^ d^-1^** |  |
| **Long-term trap deployments** |  |  |  |
| **150 - 300 m water depth** |  |  |  |
| Lomonosov Ridge | **1.5** | 0.0003 – 0.02 | Fahl and Nöthig, 2007^7^ |
| Northern Laptev Sea | **6.6** | ~ 0.002 – 0.28 | Lalande et al., 2009^S1^ |
| Fram Strait HAUSGARTEN | **2.5** | 0.0007 – 0.045 | Bauerfeind et al., 2009^S2^;  Lalande et al., 2013^S3^; 2016^S4^ |
| **2500 m** |  |  |  |
| Fram Strait HAUSGARTEN | **3.3** | 0.002 – 0.014 | Lalande et al., 2016^S4^ |
| **Short-term trap deployments** |  |  |  |
| **5 - 25 m water depth** |  |  |  |
| Northern Laptev Sea |  | 0.03 – 0.38 | Lalande et al., 2014^20^ |
| Yermak Plateau |  | 0.017 – 0.06 | Lalande et al., 2014^20^ |
| Central Arctic Ocean |  | 0.005 – 0.11 | Lalande et al., 2014^20^ |
| **300 - 500 m** |  |  |  |
| Fram Strait HAUSGARTEN |  | 0.002 – 0.015 | Lalande et al., 2011^40^ |
| **^234^Th-derived POC flux** |  |  |  |
| **2 - 200 m water depth** |  |  |  |
| Nansen/Amundsen Basin |  | 0 – 0.12 | Roca-Marti et al., 2016^10^ |
| High Arctic | **0.9** |  | Cai et al., 2010^S5^ |
| **Nitrate flux estimation** |  |  |  |
| from nitrate budget calculated export production | **1.5 - 3.0** |  | Randelhoff and Guthrie, 2016^43^ |
|  |  |  |  |
| **> 1000 m water depth** |  |  |  |
| High Arctic | **0.17 – 1.05** |  | Review by Harada, 2016 and references therein^9^ |

| **Supplementary Table 2:** Potential carbon budget; estimation for the *Phaeocystis* blooming event of about 28 days integrated for the upper 50 m water column. Particulate organic carbon (**POC**) standing stock, the contribution of *Phaeocystis* biomass in carbon units to the entire phytoplankton community (**PPC**, phytoplankton carbon) for station PS92/47 and for the N-ICE 2015 expedition (calculated averages, AVG), integrated *Phaeocystis* standing stocks and primary production rates (50 m). The estimated potential carbon export is based on the daily primary production of *Phaeocystis* for this study and the average daily carbon export rates at 100 m derived from short term sediment traps deployed under the ice for NICE 2015 multiplied by the duration of the bloom (28 days). ± indicate method error.   \|  \| **POC** standing stock  (g C m^-2^*)* \| *Phaeocystis*  contribution to **PPC**  (%) \| *Phaeocystis*  standing stock (**PPC**)  (g C m^-2^) \| *Phaeocystis*  primary production  (g C m^-2^ d^-1^) \| *Phaeocystis*  estimated potential carbon export for 28days  (g C m^-2^) \| Reference \| \| --- \| --- \| --- \| --- \| --- \| --- \| --- \| \| PS92/47 \| 9.4±0.5 \| 56% \| 1.6±0.1 \| 0.13±0.02 \| 3.8±0.2 \| This study \| \|  \|  \|  \|  \|  \|  \|  \| \| AVG  NICE 2015 \| 11.14 \| 74% \| 1.3 \| - \| 4.5 \| Calculated  from  Assmy et. al., 2017^13^ \| |
| --- | --- | --- | --- | --- | --- | --- | --- | --- | --- | --- | --- | --- | --- | --- | --- | --- | --- | --- | --- | --- | --- | --- | --- | --- | --- | --- | --- | --- |

**Supplementary References**

S1. Lalande, C., Belanger, S. & Fortier, L. Impact of a decreasing sea ice cover on the vertical export of particulate organic carbon in the northern Laptev Sea, Siberian Arctic Ocean. *Geophys. Res. Lett* **36**, L21604, (2009).

S2. Bauerfeind, E. et al. Particle sedimentation patterns in the eastern Fram Strait during 2000-2005: Results from the Arctic long-term observatory HAUSGARTEN. *Deep Sea Res. I* **56**, 1471-1487, (2009).

S3. Lalande, C., Bauerfeind, E., Nöthig, E.-M. & Beszczynska-Möller, A. Impact of a warm anomaly on export fluxes of biogenic matter in the eastern Fram Strait. *Progr. Oceanogr*. **109**, 70-77, (2013).

S4. Lalande, C. et al. Lateral supply and downward export of particulate matter from upper waters to the seafloor in the deep eastern Fram Strait, *Deep Sea Res. I* **114**, 78-89, (2016).

S5. Cai, P. et al. Low export flux of particulate organic carbon in the central Arctic Ocean as revealed by 234Th:238U disequilibrium, *J. Geophys. Res* **115**, (2010).
